# Tinnitus and hearing survey: cultural adaptation to Brazilian Portuguese^☆^

**DOI:** 10.1016/j.bjorl.2019.06.009

**Published:** 2019-07-26

**Authors:** Amanda Rodrigues Scheffer, Maria Fernanda Capoani Garcia Mondelli

**Affiliations:** a Universidade de São Paulo (USP), Faculdade de Odontologia de Bauru, Programa de Pós-Graduação em Audiologia e Terapia da Fala, Bauru, SP, Brazil; b Universidade de São Paulo (USP), Ciências da Reabilitação, Bauru, SP, Brazil; c Universidade de São Paulo (USP), Departamento de Audiologia e Terapia da Fala, Bauru, SP, Brazil

**Keywords:** Audiology, Hearing loss, Tinnitus, Questionnaires, Translating

## Abstract

**Introduction:**

Hearing loss is associated with several comorbidities and may be frequently associated with tinnitus. When patients complain of both tinnitus and hearing difficulties in audiology and otolaryngology clinics, there, is often great difficulty separating the two complaints. The tinnitus and hearing survey was specially developed for this purpose to identify the main complaint and help direct the choice of appropriate intervention.

**Objective:**

To translate and culturally adapt the tinnitus and hearing survey for the Brazilian-Portuguese.

**Methods:**

Seventy patients who had previously completed a battery of audiological diagnostic exams were invited to complete the tinnitus and hearing survey and were categorized into four groups: normal hearing without tinnitus, normal hearing with tinnitus, hearing loss without tinnitus, and hearing loss with tinnitus. Cultural adaptation of tinnitus and hearing survey followed the steps indicated by Guillemin et al. (1993), including assessment of inter-researchers’ reproducibility, internal consistency, and reliability of the instrument.

**Results:**

There were no substantial changes to the content of the tinnitus and hearing survey questions, although a few adaptations were made to two-item sound tolerance section to facilitate participants’ understanding. Internal consistency and reliability tested by Cronbach’s α was considered good for all domains. The reproducibility of the tinnitus and hearing survey was measured by the Kappa coefficient at two different moments and agreement between evaluators 1 and 2 was considered almost perfect, indicating good reproducibility.

**Conclusion:**

The tinnitus and hearing survey was culturally adapted to Brazilian Portuguese and analyzed for internal consistency, reliability, and reproducibility. Results support this questionnaire as a useful tool to help professionals differentiate the main complaint of the individual, allowing the choice of a more appropriate intervention.

## Introduction

Hearing loss can cause psychosocial impairments due to withdrawal from social interaction and occupational activities.^1^ In addition to hearing complaints, tinnitus is often reported—an auditory perception noted only by the affected individual, which can cause problems of concentration, difficulty sleeping, irritation, social withdrawal, and negative emotional reactions.^2^, ^3^, ^4^

Several factors can be associated with the onset of tinnitus, including hearing loss, metabolic, neurological, psychiatric, and otological disorders, dental problems, cardiovascular disorders, as well as drug side effects, possibly with caffeine, nicotine, and alcohol.^5^, ^6^, ^7^

The relationship between hearing loss and tinnitus has been widely cited.^8^, ^9^ According to Ferrari et al.,^10^ Sanchez et al.,^3^ and Cantley et al.,^11^ 85–96% of patients with tinnitus present some degree of hearing loss. The combination of symptoms can significantly impact a patient’s daily life.

Tinnitus affects approximately 15% of the world’s population and can be manifest independently of age.^12^ In the state of São Paulo, 22% of 1960 individuals interviewed had a tinnitus complaint, indicating this symptom is a prevalent stress factor capable of affecting the quality of life for many of these individuals. Approximately 20% of people who experience tinnitus are significantly impacted by the condition, but they cannot identify the determinants of their discomfort.^13^, ^14^

The degree of annoyance caused by tinnitus is correlated with the discomfort caused by associated hearing loss, which may justify the erroneous attribution of hearing difficulties to tinnitus by many patients who complain of tinnitus. Such a misattribution makes it essential to separate the problems caused by tinnitus from those caused by hearing loss, to more appropriately target any needed treatment.^15^, ^16^

Problems of sound tolerance also continue to be a complex phenomenon that has only recently attracted significant attention.^17^ Decreased tolerance to sound can be defined as the presence of negative reactions experienced by a person as a result of exposure to sounds that would not evoke such reactions in a listener considered to possess normal hearing.^18^

Sound-tolerance problems are more likely to occur in tinnitus-affected individuals, and the use of a biopsychosocial conceptualization of tinnitus and other behavioral medicine conditions could be helpful in understanding and treating these problems.^19^

Based on the tinnitus literature, it is possible to estimate the prevalence of decreased sound tolerance in the general population. In the study conducted by the Emory Tinnitus and Hyperacusis Center in Atlanta, 60% of patients with tinnitus were reported to have decreased sound tolerance.^17^, ^20^

Limited information is available regarding the epidemiology, mechanisms, and results of treatments for decreased sound tolerance, and, consequently, many patients with this disorder remain unassisted, suggesting necessary improvements in the diagnosis and treatment of the problems of low sound tolerance, as well as a greater number of researches that can lead to a better understanding of this problem.^17^

The Tinnitus and Hearing Survey (THS) was developed to determine how much of a patient’s complaint is related to hearing problems and how much is directly related to tinnitus, as well as to identify potential problems with decreased sound tolerance.^15^

Due to the shortage of instruments in Portuguese-Brazilian for evaluating and differentiating symptoms of hearing loss and tinnitus, the proposal of this research is to translate and adapt culturally the THS to enable hearing health professionals to identify the degree to which these two auditory symptoms impact the life of the patient, helping to identify problems of sound tolerance, providing more effective intervention, and consequently better quality of life.

## Methods

The study was developed as a non-randomized clinical trial, with the approval of the Research Ethics Committee under CAAE nº 59804216.1.0000.5417.

The patients invited to complete the questionnaire previously passed a battery of audiological diagnostic tests composed of: Tonal Audiometry, Speech Audiometry and Acoustic Immittance Measurements.

The cultural adaptation of the Tinnitus and Hearing Survey (THS) followed the steps recommended by Guillemin et al.,^21^ including translation of the English language version into Portuguese, translation from Portuguese into English (i.e., back-translation), linguistic adaptation, and revision of the grammatical and idiomatic equivalences and the evaluation of the inter-researcher reproducibility of this questionnaire. The questionnaire’s author authorization was requested for cultural adaptation.

A total of 70 patients of both sexes, with and without complaints of hearing loss and tinnitus, who met the study inclusion criteria, were interviewed between March and November 2017, following their battery of audiological diagnostic tests. To classify patients as having either hearing loss or normal hearing, mean thresholds in dB HL at 500, 1000, 2000 and 4000 Hz were used. Using the World Health Organization criteria,^21^ mean hearing thresholds ≤25 dB HL indicated normal hearing and >25 dB HL indicated hearing loss. Hearing loss was further characterized as mild (mean 26–40 dB HL), moderate (mean 41–60 dB HL), severe (mean 61–80 dB HL) and profound (mean >80 dB HL), according to the WHO^22^ recommendation.

Four groups were formed: Group 1 (normal hearing and no tinnitus) consisting of 13 women (65%) and 7 men (35%); Group 2 (normal hearing and tinnitus) consisting of 9 women (64.28%) and 5 men (35.72%); Group 3 (hearing loss and no tinnitus) consisting of 6 women (42.86%) and 8 men (57.14%); and Group 4 (hearing loss and tinnitus) consisting of 10 women (45.45%) and 12 men (54.55%).

The translation and cultural adaptation of the THS followed the five stages suggested by Guillemin et al.^21^

### Stage 1: translation

For the linguistic adaptation from English to Portuguese, three translators-interpreters, fluent in both languages, unknown to each other, and who had no prior contact with the questionnaire, individually and confidentially produced a first version of the Portuguese-Brazilian questionnaire. Thus, three translated versions of the THS were generated in this first stage.

### Stage 2: overview

In Stage 2, a review group composed of three audiologists fluent in the English language analyzed the three documents resulting from the first stage and, together, reconciled the differences found between translations. The best expressions and words were selected, as well as the choice of appropriate terms in all items, in order to adapt the text to the Brazilian culture, making it understandable by this population.

From this process, a single questionnaire was generated, the first version of THS in Brazilian-Portuguese.

### Stage 3: back-translation

In the back-translation stage, a copy of the first version of THS in Portuguese-Brazilian was sent to three new, knowledgeable, and fluent translators of the English language. These new translators did not have knowledge of the original text so there was no influence of vocabulary. Thus, a new English version of THS was generated.

### Stage 4: final reviewer

For the fourth stage, a linguistic speech therapist, fluent in the English language was invited to be the final reviewer and tasked with analyzing the language used in the texts, as well as the reverse translations in order to resolve any discrepancies. In this way, the final version of the THS in Brazilian-Portuguese was constituted (Appendix 1 in Supplementary material).

### Stage 5: application of the questionnaire

In the fifth stage, the 70 patients were interviewed individually, with and without tinnitus complaints. During the application of the questionnaire the researchers took notes of any difficulties understanding items, or of the specific terms that participants had doubts about. If these difficulties occurred with more than 20% of interviewees on the same question, it would be submitted as a new translation.

### Evaluation

#### Consistency, reliability and reproducibility

After completing the five stages of translation and cultural adaptation of the instrument, internal consistency and reliability of the instrument were evaluated, using data from the questionnaire applications answered by the 70 patients.

Two interviews were made with each participant, involving two researchers using the same questionnaire. All questions were required to be answered, and the respondent could elucidate concerns at any time during the interview and/or ask for any of the questions to be repeated.

To confirm inter-researcher reproducibility, the questionnaire was applied to patients by Interviewer 1 and on the same day at an interval of approximately 20 min by a second interviewer (Interviewer 2) in order to verify if the patients’ responses were the same.

It was not possible to verify the results using intra-researcher reproducibility, as all the questions asked related to the previous week’s application of the questionnaire. As the symptoms of tinnitus can change over time, this made it impractical to apply the questionnaire over a two-week period.

### Form of analysis

The data were entered into Microsoft Excel by group, tabulated, and described according to the descriptive statistical analysis of the discrete and continuous quantitative variables and nominal and ordinal qualitative variables.

Data analysis was performed using the STATISTICA program (StatSoft Inc., Tulsa, USA), based on inductive statistics that allows the researcher to construct propositions about the studied population.

For the descriptive analysis, the mean and standard deviation of the numerical variables were utilized.

For the internal consistency test, the Cronbach α coefficient was used. The reliability index for the Cronbach α considered as good was 80–90% and very good above 90%.^23^ In order to compare the qualitative inter-researcher variable, we used the Kappa Concordance Analysis and adopted the values from 0.81 to 1.00 as a near perfect comparison.^24^

## Results

For the cultural adaptation process the sample consisted of 70 patients, 36 females (51.4%) and 34 males (48.6%), divided into the four groups. The mean age of the sample was 51.6, with a standard deviation of 16.7, and ranged from 21 (minimum) to 87 (maximum) years of age. Table 1 shows the sample description.

**Table 1:** Sample description.

	Females	Males	Total
Total sample	36 (51.4%)	34 (48.6%)	70 (100%)
Age (SD)	50.8 (15.6)	52.4 (18.1)	51.6 (16.7)
G1	13 (65%)	7 (35%)	20 (28.6%)
Age (SD)	46.2 (14.8)	28.6 (8.6)	40.1 (15.4)
G2	8 (57.1%)	6 (42.9%)	14 (20%)
Age (SD)	62.2 (5.6)	66.6 (14.0)	44.9 (16.0)
G3	6 (42.9%)	8 (57.1%)	14 (20%)
Age (SD)	43.3 (19.0)	46.1 (14.5)	64.1 (9.9)
G4	9 (40.9%)	13 (59.1%)	22 (31.4%)
Age (SD)	52.2 (16.5)	62.6 (9.5)	58.4 (13.5)

SD, standard deviation; G, group.

There were no major changes in the content of the THS items; only a few adaptations in the Sound Tolerance section were made to facilitate correct understanding. Regarding the comment (not one of the items), “If sounds are too loud for you while wearing hearing aids, please tell your audiologist” the term “audiologist” has been translated to “fonoaudiólogo”, because in Brazil there is no distinction between the professions of Audiologist and Speech Therapist as there is in the United States where the questionnaire was developed.

There was also the need to cite examples of the types of sounds not considered commonly problematic, as most patients reported discomfort to sounds considered loud by anyone, for example loud music or car engine noise.

In the application stage, there was no need to review any item. The THS did not present technical terms that made it difficult to understand the translation and cultural adaptation, since the minimum level of difficulty, set at 20% for the reformulation of the questions, was not reached. The modifications made by the review committee were made with the purpose of adjusting the instrument to the Brazilian culture.

The internal consistency and reliability tested by Cronbach’s α were considered good for all domains (99%), indicating that all questions within each domain were reliable (Table 2).

**Table 2:** Internal consistency (Cronbach's α) and Reliability evaluator 1 and evaluator 2 (Intraclass correlation coefficient).

Domain	Number of Questions	Cronbach’s α (n = 70)	Intraclass correlation (95% IC) (n = 70)
Tinnitus	4	0.998	0.995 (0.992‒0.997)
Hearing loss	4	0.999	0.998 (0.997‒0.998)

IC, intraclass correlation.

The reproducibility of the questionnaire was measured by the Kappa coefficient in two different moments (Table 3). As shown in Table 3, there was agreement between 0.81 and 1.00 considered almost perfect between evaluators 1 and 2, indicating good reproducibility.

**Table 3:** Inter-researcher reproducibility in qualitative variables (Kappa).

Domain	Kappa coefficient (n = 70)	Strength of agreement
Tinnitus (R1–R2)		
Q1	0.942	Almost perfect (0.81–1.000)
Q2	0.967	Almost perfect (0.81–1.000)
Q3	1.000	Almost perfect (0.81–1.000)
Q4	0.968	Almost perfect (0.81–1.000)
Hearing loss (R1‒R2)		
Q1	1.000	Almost perfect (0.81–1.000)
Q2	0.979	Almost perfect (0.81–1.000)
Q3	0.962	Almost perfect (0.81–1.000)
Q4	0.959	Almost perfect (0.81–1.000)
Sound tolerance (R1–R2)		
Q1	0.961	Almost perfect (0.81–1.000)

R1, Researcher 1; R2, Researcher 2; Q, question.

## Discussion

In recent years, tinnitus has been studied by many health professionals, as it is a symptom that can cause great annoyance in individuals and thereby, new tools are needed to reliably assess this symptom^25^ and to enable its classification and differentiation from other auditory complaints.

To accomplish this classification and differentiation, several instruments have been created to assist in identifying the most appropriate intervention for each patient. In Brazil, however, only the THI was validated for the Brazilian population, because it is a questionnaire that is easily applied and interpreted and for evaluating emotional aspects that cause interference in the individual’s social and emotional life.^26^, ^27^

In clinical practice, it is noted there is difficulty in differentiating the tinnitus complaint from other auditory disorders, so that many individuals erroneously attribute their auditory complaints to tinnitus,^15^, ^26^ making it is necessary to use other methods to measure and distinguish which problem most affect the quality of life of these patients: hearing loss or tinnitus.

The Tinnitus and Hearing Survey was chosen to be translated and adapted to Brazilian-Portuguese because it is a simple questionnaire, easy to apply, and specific to distinguishing one auditory problem from the other, enabling the professional to provide appropriate intervention and directed to the complaint of this population and beyond easy understanding by the patient, the application of the THS questionnaire is applied in a short time, ratifying the importance of this instrument at the time of clinical anamnesis. After translation and cultural adaptation, the THS remained with the same number of items from the original instrument.

Although there are several methods for translation and cultural adaptation to support the researcher, for the translation and cultural adaptation of THS, we chose Guillemin et al.’s^21^ proposition, which consists of five stages: translation, synthesis, back-translation, review committee, and application, which is an internationally recognized method of cultural adaptation used by several authors, such as the translation and cultural adaptation of the THI questionnaire,^27^ the Dutch version of FaCE Scale,^28^ the Korean version of WHOQOL-DIS,^29^ among others.

It is important to distinguish the terms “adaptation” and “translation”. The term translation is used more often because it is the first part of the process of cultural adaptation and involves the cultural adjustment of the instrument and the passage from the original language to the target language. However, the process of cross-cultural adaptation involves the development of versions of an evaluation instrument that are equivalent to the original but at the same time linguistically and culturally adapted to a different context from the original.^30^

During the application of THS there was no doubt about the questions in sections A: Tinnitus and B: Hearing. In section C: Sound Tolerance, no problem of understanding was reported by the participants; however, the answers given did not match the problems that the question referred to, since most of the interviewees reported discomfort for sounds considered to be commonly high. In this way there was a need to cite examples so that they could understand what types of sounds we wanted to investigate with this item.

The problems of sound tolerance are still considered a complex and evasive phenomenon, and only recently have attracted greater attention.^17^

The THS helps the professional to identify problems such as misophonia and hyperacusis in patients, but it is essential that the professional understands and differentiates these symptoms to clarify them adequately to the individual, since for many years, the problems of sound tolerance have been underestimated and not fully investigated and the one who suffers from this discomfort, not knowing which professional to resort to and ended up with engender strategies to live with it, not receiving in this way the appropriate treatment and guidelines to deal with this problem.^17^, ^31^

The study by Henry et al.^15^ showed that research participants often seemed confused when they were approached about items of sound tolerance, giving answers that did not accurately represent their experience with this problem, making it clear that many people do not know what a sound tolerance problem is.

This lack of information justifies the unknowledge by the interviewees of the types of sounds we were referring in the item about sound tolerance and the reason for the necessity to add examples in the final item. Therefore, it is important to emphasize that monitoring, as well as constant and adequate counseling, are essential for overcoming the difficulties faced by this population.^32^

Instructions for the application of THS are equally important to guide the decision when dealing with patient-reported problems. They are essential to clarify individuals’ doubts and help discern hearing complaints from tinnitus complaints.

The THS proved to be a reliable questionnaire when comparing the original version of THS^15^ and the recent validation for the Polish.^33^ In both studies the measure of internal consistency and reproducibility was above 0.76 and classified as good to excellent. In his review on cultural validation and adaptation Arafat et al.^30^ shows that almost all articles evaluated the internal consistency of reliability by means of Cronbach's α measure with a level of ≥0.70.

To evaluate the reproducibility of THS, the Kappa coefficient was used, which showed that there was good reproducibility among researchers. The test-retest execution to verify good reproducibility is justified because in this way there is certainty that the instrument is stable and that it can be applied in any period and by any person and will provide concise results, as shown by a review of 78 articles.^30^

The questionnaire was applied by the interviewer of number 1 and the same day by interviewer number 2, since this refers to the previous week to which it is applied, which would impair the results of reproducibility if applied as the literature suggests. Therefore, it was not possible to perform the test-retest with the interval suggested by the literature.

The disadvantage of choosing a short time between test and retest is that individuals can remember the answers provided in the first application^33^; however, comparing the statistical results shown in this research with the results of other versions of the questionnaire, this short reapplication time did not seem to influence the outcome, since the statistical results of these surveys were similar.

For the patient with tinnitus, results of an audiometric evaluation, combined with application of THS will normally provide all of the information needed to determine if the person needs tinnitus-specific intervention,^15^ in this way we believe the Brazilian THS is, despite being a brief questionnaire, capable of helping to differentiate how much of the problems reported by the patient, may have applicability both as mediator to choose the best treatment, and as a parameter of clinical evolution and therapeutic monitoring in patients who suffer with tinnitus.

## Conclusion

The tinnitus and hearing survey was culturally adapted to Brazilian Portuguese, and its simple and easy-to-understand format is of broad applicability. Analyses of internal consistency, reliability, and reproducibility revealed that the questionnaire can be a useful tool to help the professional to differentiate the main complaint of the individual, allowing the choice of a more adequate intervention, as well as to follow its evolution during treatment.

## Conflicts of interest

The authors declare no conflicts of interest.

## References

[1] Lessa A., Costa M., Becker K., Vaucher A. (2010). Satisfaction of hearing aids users with hearing loss of severe and deep degree. Int Arch Otorhinolaryngol.

[2] Sanchez T., Zanato A., Bittar R., Bento R. (1997). Controversies about the physiology of tinnitus. Int Arch Otorhinolaryngol.

[3] Sanchez T., Medeiros Í., Levy C., Ramalho J., Bento R. (2005). Tinnitus in normally hearing patients: clinical aspects and repercussions. Rev Bras Otorrinolaringol.

[4] Dias A., Cordeiro R., Corrente J. (2006). Tinnitus annoyance assessed by the Tinnitus Handicap Inventory. Rev Saúde Públ.

[5] Knobel K. (2001). http://fonoesaude.tripod.com/extras/monografia.PDF.

[6] Mondelli M., Rocha A. (2011). Correlation between the audiologic findings and buzz disturbing. Int Arch Otorhinolaryngol.

[7] Montazeri K., Mahmoudian S., Razaghi Z., Farhadi M. (2017). Alterations in auditory electrophysiological responses associated with temporary suppression of tinnitus induced by low-level laser therapy: a before-after case series. J Lasers Med Sci.

[8] Langguth B., Landgrebe M., Schlee W., Schecklmann M., Vielsmeier V., Steffens T. (2017). Different patterns of hearing loss among tinnitus patients: a latent class analysis of a large sample. Front Neurol.

[9] Schlee W., Shekhawat G. (2017). What does tinnitus have to do with hearing loss?. Front Young Minds.

[10] Ferrari G., Sanchez T., Bernardi A. (2003). Tinnitus control through the use of ear hearing aids. Rev CEFAC.

[11] Cantley L., Galusha D., Cullen M., Dixon-Ernst C., Tessier-Sherman B., Slade M. (2014). Does tinnitus, hearing asymmetry, or hearing loss predispose to occupational injury risk?. Int J Audiol.

[12] Hoffman H., Reed G., Snow J. (2004). Tinnitus: theory and management.

[13] Pinto P., Sanchez T., Tomita S. (2010). The impact of gender, age and hearing loss on tinnitus severity. Braz J Otorhinolaryngol.

[14] Oiticica J., Bittar R. (2015). Tinnitus prevalence in the city of São Paulo. Braz J Otorhinolaryngol.

[15] Henry J., Griest S., Zaugg T., Thielman E., Kaelin C., Galvez G. (2015). Tinnitus and hearing survey: a screening tool to differentiate bothersome tinnitus from hearing difficulties. Am J Audiol.

[16] Teixeira A., Benin L., Lessa A., Rosito L., Walbrohel Í., Picinini T. (2016). Chronic tinnitus: study in subjects with and without hearing loss. ConScientiae Saúde.

[17] Jastreboff M., Jastreboff P. (2014). Treatments for decreased sound tolerance (hyperacusis and misophonia). Semin Hear.

[18] Aazh H., McFerran D., Salvi R., Prasher D., Jastreboff M., Jastreboff P. (2014). Insights from the first international conference on hyperacusis: causes, evaluation, diagnosis and treatment. Noise Health.

[19] Cash T.V. (2015). http://scholarscompass.vcu.edu/cgi/viewcontent.cgi?article=5122&context=etd.

[20] Jastreboff M., Jastreboff P. (2002). Decreased sound tolerance and Tinnitus Retraining Therapy (TRT). Australia New Zealand J Audiol.

[21] Guillenim F., Bombardie C., Beaton D. (1993). Cross-cultural adaptation of health-related quality of life measures: literature review and proposed guidelines. J Clin Epidemiol.

[22] (2019). Grades of hearing impairment.

[23] Pestana M., Gageiro J. (2014). http://www.silabo.pt/conteudos/7752_PDF.pdf.

[24] Landis J., Koch G. (1977). The measurement of observer agreement for categorical data. Biometrics.

[25] Müller K., Edvall N., Idrizbegovic E., Huhn R., Cima R., Persson V. (2016). Validation of online versions of Tinnitus Questionnaires Translated into Swedish. Front Aging Neurosci.

[26] Ferreira P., Cunha F., Onishi E., Branco-Barreiro F., Ganança F. (2005). Tinnitus handicap inventory: cross-cultural adaptation to Brazilian Portuguese. Pró-Fono.

[27] Dobie R., Snow J. (2004). Tinnitus: theory and management.

[28] Kleiss I., Beurskens C., Stalmeier P., Ingels K., Marres H. (2015). Quality of life assessment in facial palsy: validation of the Dutch Facial Clinimetric Evaluation Scale. Eur Arch Otorhinolaryngol.

[29] Lee K., Jang H., Choi H. (2017). Korean translation and validation of the WHOQOL-DIS for people with spinal cord injury and stroke. Disabil Health J.

[30] Arafat S.M.Y., Chowdhury H.R., Qusar M.S., Hafez M. (2016). Cross-cultural adaptation and psychometric validation of research instruments: a methodological review. J Behav Health.

[31] Cordeiro B., Souza G., Mendes C. (2016). Misophonia: when certain sounds drive people crazy. Rev Ciênc Méd Biol.

[32] Ribas A., Kozlowsk L., Almeida G., Marques J., Silvestre R., Mottecy C. (2019). Quality of life: comparing results in elderly with and without presbyacusis. Rev Bras Geriatr Gerontol.

[33] Raj-Koziak D., Gos E., Rajchel J., Piłka A., Skarżyński H., Rostkowska J. (2017). Tinnitus and Hearing Survey: a polish study of validity and reliability in a clinical population. Audiol Neurootol.

