# Supplementary material for: Tinnitus and hearing survey: cultural adaptation to Brazilian Portuguese
Source: Braz J Otorhinolaryngol. 2019 Jul 26;87(1):28–34. doi: 10.1016/j.bjorl.2019.06.009 (PMC9422566; doi:10.1016/j.bjorl.2019.06.009)
Supplement: Supplementary file 1 [file mmc1.docx]

**Appendix 1** Final version of Tinnitus and Hearing Survey – Brazilian-Portuguese.

| **A - Zumbido** | **Não, não é um problema** | | **Sim, é um problema pequeno** | **Sim, é um problema moderado** | | **Sim, é um problema grande** | **Sim, é um problema muito grande** | |
| --- | --- | --- | --- | --- | --- | --- | --- | --- |
| Na última semana, o zumbido me impediu de dormir. | 0 | | 1 | 2 | | 3 | 4 | |
| Na última semana, o zumbido me impediu de me concentrar na leitura. | 0 | | 1 | 2 | | 3 | 4 | |
| Na última semana, o zumbido me impediu de relaxar. | 0 | | 1 | 2 | | 3 | 4 | |
| Na última semana, não consegui tirar o zumbido da minha cabeça. | 0 | | 1 | 2 | | 3 | 4 | |
| Total de cada coluna |  | |  |  | |  |  | |
| Total geral |  | | | | | | | |
|  | | | | | | | | |
| **B - Audição** | **Não, não é um problema** | | **Sim, é um problema pequeno** | **Sim, é um problema moderado** | | **Sim, é um problema grande** | **Sim, é um problema muito grande** | |
| Na última semana, eu não consegui entender o que as pessoas estavam dizendo em um lugar barulhento ou com muitas pessoas. | 0 | | 1 | 2 | | 3 | 4 | |
| Na última semana, eu não consegui entender o que as pessoas estavam dizendo na TV ou nos filmes. | 0 | | 1 | 2 | | 3 | 4 | |
| Na última semana eu não entendi pessoas que falavam baixo. | 0 | | 1 | 2 | | 3 | 4 | |
| Na última semana, eu não consegui entender o que estava sendo dito em uma conversa com várias pessoas juntas (ou conversa em grupo). | 0 | | 1 | 2 | | 3 | 4 | |
| Total de cada coluna |  | |  |  | |  |  | |
| Total geral |  | | | | | | | |
|  | | | | | | | | |
| **C - Tolerância ao som** | **Não, não é um problema.** | | **Sim, é um problema pequeno.** | **Sim, é um problema moderado** | | **Sim, é um problema grande.** | **Sim, é um problema muito grande.** | |
| Na última semana, os sons estavam muito altos ou desconfortaveis para mim enquanto eles pareciam confortáveis para as pessoas em volta* (Pessoas mastigando, papel amassando) | 0 | | 1 | 2 | | 3 | 4 | |
| Se você respondeu 1, 2, 3 ou 4 à declaração acima: | | | | | | | | |
| *Por favor, liste dois exemplos de sons que são muito altos ou desconfortáveis para você, mas parecem normais para os outros | |  | | | | | | |
|  |  |  | | | | | | |
|  | | * Se os sons estiverem desconfortáveis durante o uso do aparelho auditivo, favor consultar seu fonoaudiólogo. | | | | | | |
| Apenas para uso do profissional (II): | | M | | | H | | | N |
|  | | | | | | | | |
| **Instruções para uso do Questionário Zumbido e Audição (THS).** | | | | | | | | |
| **Categoria A e B** | | | | | | | | |
| Os quatro itens da categoria A (zumbido) descrevem problemas comuns relacionados ao zumbido que não estão relacionados a problemas auditivos. Os quatro itens da categoria B (audição) descrevem problemas auditivos comuns que não são causados pelo zumbido. O passo a passo para o uso do THS irá determinar colaborativamente se a intervenção para o zumbido é desejável e apropriada. Siga as instruções a baixo: | | | | | | | | |
| 1. Explique que a intervenção ao zumbido pode ajudar com os problemas na categoria A; | | | | | | | | |
| 2. Explique que a intervenção ao zumbido pode não ajudar em nenhum dos problemas listados na categoria B; | | | | | | | | |
| 3. Descreva o que é necessário para intervenção do zumbido (logística, custo, etc.); | | | | | | | | |
| 4. Esteja disponível para responder a perguntas ou preocupações sobre a intervenção do zumbido que será oferecida, ou sobre o zumbido em geral; | | | | | | | | |
| 5. Permita que o paciente decida se quer ou não participar na intervenção | | | | | | | | |
| O uso da nota de corte para determinar a candidatura do paciente para a intervenção ao zumbido não será utilizada, pois ela não leva em conta todos os fatores da vida do paciente. | | | | | | | | |
| O uso mais eficaz do THS é como ferramenta rápida e eficiente para diferenciar os problemas de audição de problemas com zumbido, o que permite ao clinico intervenções disponíveis para o problema enfrentado pelo paciente. | | | | | | | | |
| O paciente pode então decidir se alguma das intervenções que estão sendo oferecidas é uma boa opção para seu estilo de vida, e para os problemas que desejam abordar. | | | | | | | | |
|  | | | | | | | | |
| **Categoria C** | | | | | | | | |
| Problemas de tolerância ao som são frequentemente relatados por pacientes com zumbido. Os dois itens na categoria C (Tolerância ao Som) podem ser usados ​​para auxiliar o clínico a desenvolver uma impressão inicial quanto à existência e ao tipo de problema de tolerância ao som. | | | | | | | | |
| O item 1 é usado para detectar a existência de um problema de tolerância ao som. Qualquer resposta diferente de zero indica algum nível de dificuldade em tolerar o som. | | | | | | | | |
| O item 2 pretende extrair exemplos do paciente (que o profissional discutirá com o paciente) para: (1) garantir que o paciente realmente está experimentando um problema de tolerância ao som (e não outra coisa); (2) informar a opinião do clínico quanto ao tipo de problema de tolerância ao som. | | | | | | | | |
| (1) sons que seriam muito altos para qualquer um (por exemplo, fogos de artifícios, sirene nas proximidades); (2) referências a problemas de tolerar multidões ou outras situações por razões diferentes da tolerância ao som (por exemplo, hipervigilância ou outros sintomas de Transtorno de Stress Pós-Traumático, dificuldade em entender o que as pessoas estão dizendo); (3) queixas de usuários de aparelhos auditivos que apenas têm problemas para tolerar sons que são comumente problemáticos para os mesmos (por exemplo, talheres ou pratos batendo, papel sendo amassado). | | | | | | | | |
| Após a discussão dos exemplos, se o paciente demonstrar que apresenta problemas com a tolerância de sons que a maioria das pessoas pode tolerar sem dificuldade, o clinico irá trabalhar com a possibilidade de que a intolerância do som pode ser por hiperacusia, misofonia ou a combinação dos dois. Use as definições abaixo para orientações durante a conversa com o paciente, utilizando os exemplos apresentados pelo paciente. | | | | | | | | |
| **Hiperacusia = desconforto físico causado pelo som em níveis que são confortáveis para a maioria das pessoas.** Com hiperacusia, todos os sons são desconfortáveis quando atingem um certo nível de sonoridade, que varia de pessoa a pessoa com hiperacusia. | | | | | | | | |
| **Misofonia = reações emocionais ao som.** Com misofonia, não é a intensidade do som que causa desconforto (como no caso da hiperacusia), mas sim a reação emocional causada pelo som que torna a experiência desconfortável. É comum a uma pessoa com misofonia achar sons fracos particularmente desconfortáveis, mas achar outros sons no mesmo nível aceitável. | | | | | | | | |
| “Apenas para uso do profissional (II)” para se referir à impressão do entrevistador se a pessoa apresenta ou não problema a intolerância ao som. | | | | | | | | |
| Utilizar o M, quando houver suspeita de Misofonia. | | | | | | | | |
| Utiliza o H, quando houver suspeita de Hiperacusia. | | | | | | | | |
| E utilizar M e H, quando houver suspeita de ambos. | | | | | | | | |
| Se não houver condição suspeita, utilizar o N. | | | | | | | | |
